# A non-proteolytic role for ubiquitin in deadenylation of *MHC-I* mRNA by the RNA-binding E3-ligase MEX-3C

**DOI:** 10.1038/ncomms9670

**Published:** 2015-10-16

**Authors:** Florencia Cano, Radu Rapiteanu, G. Sebastiaan Winkler, Paul J. Lehner

**Affiliations:** 1 Cambridge Institute for Medical Research, University of Cambridge, Cambridge Biomedical Campus, Hills Road, Cambridge CB2 0XY, UK; 2 Laboratory of Lymphocyte Signalling and Development, Babraham Institute, Cambridge CB22 3AT, UK; 3 School of Pharmacy and Centre for Biomolecular Sciences, University of Nottingham, University Park, Nottingham NG7 2RD, UK

## Abstract

The regulation of protein and mRNA turnover is essential for many cellular processes. We recently showed that ubiquitin—traditionally linked to protein degradation—directly regulates the degradation of mRNAs through the action of a newly identified family of RNA-binding E3 ubiquitin ligases. How ubiquitin regulates mRNA decay remains unclear. Here, we identify a new role for ubiquitin in regulating deadenylation, the initial and often rate-limiting step in mRNA degradation. MEX-3C, a canonical member of this family of RNA-binding ubiquitin ligases, associates with the cytoplasmic deadenylation complexes and ubiquitinates CNOT7(Caf1), the main catalytic subunit of the CCR4-NOT deadenylation machinery. We establish a new role for ubiquitin in regulating *MHC-I* mRNA deadenylation as ubiquitination of CNOT7 by MEX-3C regulates its deadenylation activity and is required for *MHC-I* mRNA degradation. Since neither proteasome nor lysosome inhibitors rescued MEX-3C-mediated *MHC-I* mRNA degradation, our findings suggest a new non-proteolytic function for ubiquitin in the regulation of mRNA decay.

## Abstract (web summary)

mRNA deadenylation, the first step in regulated degradation, is mediated by the action of the CCR4-NOT and PAN2-PAN3 complexes. Here the authors show that the RNA-binding E3 ubiquitin-ligase MEX-3C associates with the CCR4-NOT complex and ubiquitinates the catalytic subunit CNOT7 to regulate its deadenylation activity.

## Graphical abstract

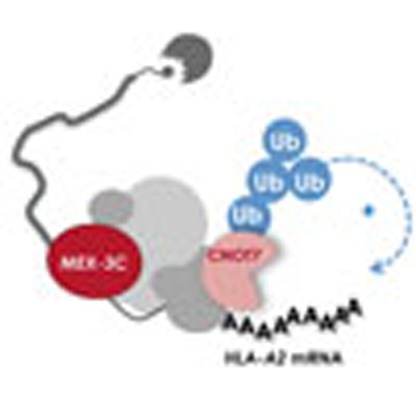

Messenger RNA (mRNA) turnover plays a critical role in the regulation of the majority of cellular processes. Up to 50% of the changes seen in gene expression are estimated to occur at the level of mRNA stability^1^,^2^, although how mammalian mRNA decay is regulated remains poorly understood. Although ubiquitin is traditionally associated with protein degradation, we recently identified a role for ubiquitin in the degradation of mRNA^3^. Of the more than 600 E3 ubiquitin ligases described, at least 15 contain an RNA-binding domain^4^ in addition to the RING domain, which defines the largest family of E3 ligases and is required for the ubiquitination reaction. In a small interfering RNA (siRNA) ubiquitome screen, we identified MEX-3C, a canonical member of this novel family of RNA-binding ubiquitin E3-ligases, which regulates the cell surface expression of major histocompatibility complex (MHC) class I proteins, via the post-transcriptional regulation of *MHC-I* mRNA. MEX-3C therefore provides a direct link between ubiquitination and mRNA degradation^3^.

The four members of the mammalian MEX-3 gene family (MEX-3A–D) each contain two RNA-binding KH domains and a ubiquitin E3-ligase RING domain^5^. This family has arisen by gene duplication from the MEX-3 orthologue in *Caenorhabditis elegans,* which also has two RNA-binding KH domains but lacks the RING domain. MEX-3C binds the 3′ untranslated region (UTR) of its target mRNA *HLA-A2* (an MHC-I allotype) through its KH domains and together with its cargo mRNA shuttles from the nucleus to the cytosol^3^,^5^. This *HLA-A2* mRNA *s*ubstrate bound to MEX-3C cannot be translated into protein, a function conserved with its *C. elegans* MEX-3 ancestor that also binds mRNA but lacks the RING domain^6^. However, although the *C. elegans* MEX-3 inhibits translation of its substrate mRNA^6^, MEX-3C not only inhibits translation but also induces the degradation of its target mRNA in a ubiquitin-dependent manner^3^. In the absence of a RING domain, MEX-3C is still able to inhibit substrate translation, but there is an absolute requirement for the RING domain, and therefore E3-ubiquitin ligase activity, for *HLA-A2* mRNA degradation. RINGless MEX-3C therefore behaves more like its *C. elegans* ancestor, in that its substrate *HLA-A2* mRNA is effectively sequestered and not translated, but is no longer degraded.

Eukaryotic mRNAs are protected from decay at their 5' and 3' ends by the cap and poly(A) tail, respectively. The degradation of mRNAs starts with the removal of the poly(A) tail by a process called deadenylation. This process is mediated by the concerted action of two complexes, namely CCR4-NOT and PAN2-PAN3. Studies in several model organisms show that deadenylation is a rate-limiting step for mRNA degradation^7^, and its impaired regulation is associated with a variety of cellular conditions in mammalian cells^8^. However, how mammalian deadenylation is regulated remains poorly understood.

Our characterization of MEX-3C’s E3 ligase activity in the regulation of mRNAs decay now establishes a new role for ubiquitin in the regulation of deadenylation. Here, we show that MEX-3C associates with different members of the cytoplasmic deadenylation complexes and ubiquitinates CNOT7, the main catalytic subunit of the CCR4-NOT deadenylation machinery. Ubiquitination of this subunit (CNOT7) by MEX-3C regulates its deadenylation activity and is required for *HLA-A2* mRNA degradation. Moreover, since neither proteasome nor lysosome inhibitors, nor the use of ubiquitin mutants that prevent the formation of protein degradation signalling K11- and K48-linked chains, rescued MEX-3C-mediated mRNA degradation, our findings point to a new non-proteolytic function for ubiquitin in the regulation of mRNA decay.

## Results

### MEX-3C interacts with the major deadenylation complexes

**Figure 1:**
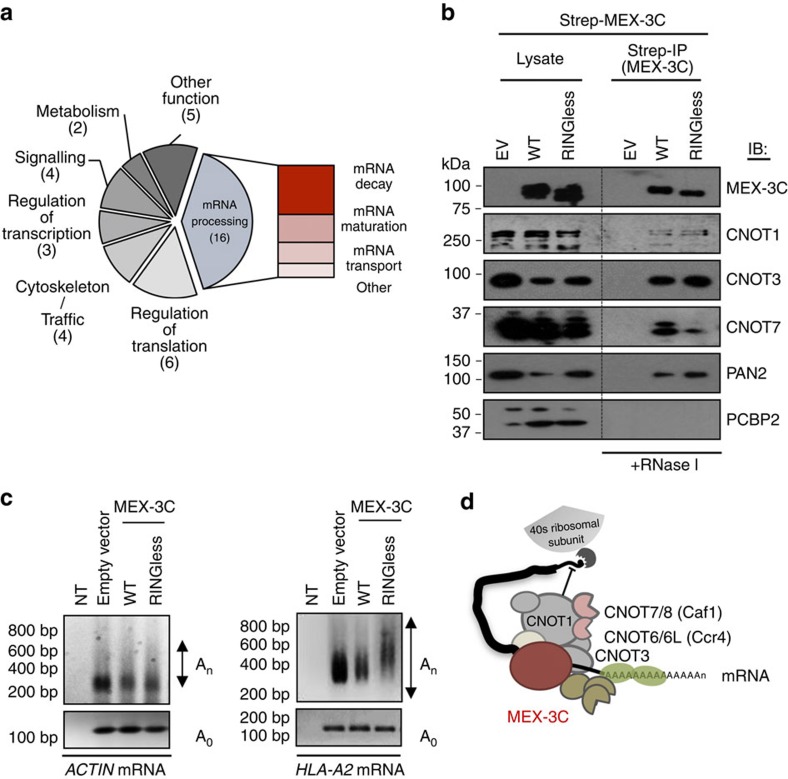
MEX-3C interacts with the major cytoplasmic deadenylation complexes and promotes the shortening of its target mRNA poly(A) tail through its ubiquitin ligase activity. (**a**) Gene ontology annotation of proteins binding RINGless MEX-3C. Number of unique proteins is in brackets. Identity of interacting proteins is shown in Supplementary Table 1. (**b**) MEX-3C interacts with the two mammalian deadenylation complexes CCR4-NOT and PAN2-PAN3. IB, immunoblot. The KH containing RNA-binding protein, PCBP2, was used as negative control. Immunoprecipitations were done in the presence of 20 U ml^−1^ RNase-I. EV, empty vector/GFP. (**c**) Shortening of *HLA-A2* mRNA’s poly(A) tail is promoted by MEX-3C’s ubiquitin ligase activity. RT–PCR-based Poly(A) tail length assay (PAT) for *HLA-A2* mRNA in control (EV/GFP), wild-type or RINGless MEX-3C-expressing (FACS sorted) cells (right panel). *ACTIN* mRNA was assayed as control (left panel) and Ao PCR controls for loading. Ao: refers to PCR products using primers to amplify the last 100–200 bp of the 3'UTR, excluding the poly(A) tail. NT, no template. (**d**) Schematic representation of the different components of the mammalian CCR4-NOT and PAN2-PAN3 deadenylation complexes.

To establish the ubiquitin-related mechanism responsible for MEX-3C-mediated degradation of mRNAs, we first sought to identify MEX-3C-binding partners involved in mRNA degradation, or potential ubiquitination substrates. We performed a pull-down with RINGless MEX-3C expressed in HEK293T cells in the presence of RNAse-I, followed by mass spectrometry analysis (Supplementary Table 1). The rationale behind this experiment was that the RINGless mutant form of MEX-3C, which is unable to ubiquitinate, should act as a substrate trap and remains bound to its ubiquitination substrates. Analysis of the RINGless MEX-3C interactome reflected MEX-3C’s involvement in different stages of RNA metabolism, primarily mRNA processing, with an emphasis on mRNA degradation (Fig. 1a).

The cytosolic degradation of eukaryotic mRNAs requires the initial shortening of the 3′-poly(A) tail (deadenylation) and subsequent removal of the 5′-cap (decapping). Deadenylation is mediated by the concerted action of two complexes, CCR4-NOT and PAN2-PAN3, both of which were found in association with MEX-3C and subsequently confirmed in immunoprecipitated (IP) blots following RNAse-I treatment (Supplementary Table 1, Fig. 1b and Supplementary Fig. 1a for control IP). PolyA-binding protein interacts with MEX-3C through its RNA cargo^5^. To control for effective RNAse-I treatment, we showed that under the experimental conditions used, our mass spectrometry analysis did not identify PolyA-binding protein bound to MEX-3C (Supplementary Table 1).

The identification of MEX-3C bound to the cytosolic deadenylation complexes is especially relevant as, despite deadenylation of mRNAs being the initial and often rate-limiting step in mRNA degradation^9^, little is known about how these deadenylases are regulated in mammalian cells. To determine whether MEX-3C promotes the deadenylation of its endogenous model substrate, *HLA-A2* mRNA *in-vivo,* we used an reverse transcription–PCR-based assay to measure the length of *HLA-A2* mRNA poly(A) tail in MEX-3C-expressing cells (Fig. 1c) following fluorescence-activated cell sorting (FACS) (Supplementary Fig. 1b). MEX-3C promotes the shortening of the poly(A) tail of *HLA-A2* mRNA (Fig. 1c right panel), but not of the *ACTIN* control mRNA (Fig. 1c left panel); an activity that requires its ubiquitin-ligase activity as it is not seen with its RINGless mutant form (Fig. 1c). Similar results were obtained for *FF-Luc-HLA-A2 3′UTR* reporter mRNA^3^. Taken together, these results led us to hypothesize that MEX-3C’s E3 ligase activity controls mRNA decay through the regulation of deadenylation (Fig. 1d).

**Figure 2:**
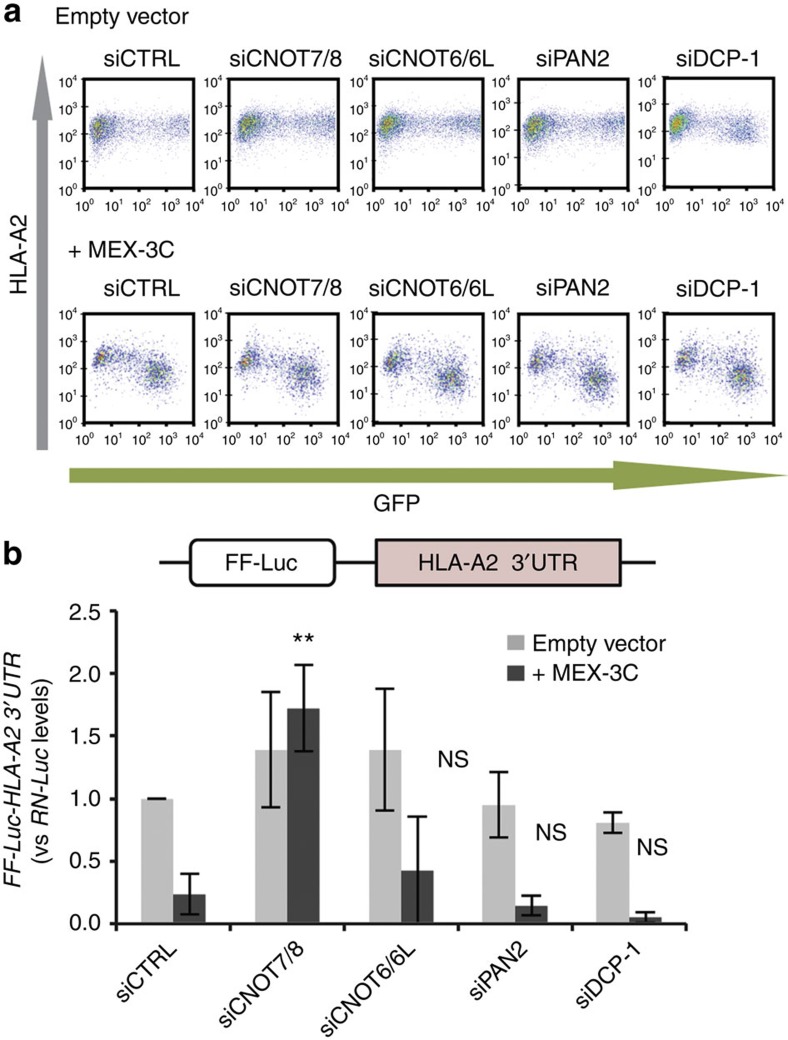
MEX-3C-mediated degradation of *FF-Luc-HLA-A2-*3′*UTR* reporter mRNA is rescued by the depletion of CNOT7/8(Caf1), the major catalytic component of the CCR4-NOT deadenylation complex. (**a**) Depletion of the catalytic components of the deadenylation complexes cannot rescue MEX-3C’s downregulation of HLA-A2 expression. Flow cytometric analysis of cell surface HLA-A2 levels in siRNA-treated HEK293T cells expressing wtMEX-3C or empty vector. GFP is a surrogate marker for MEX-3C expression. For quantification of HLA-A2 protein and mRNA levels, see Supplementary Fig. 2a–b. (**b**) Depletion of CNOT7/8(Caf1) rescues MEX-3C-mediated degradation of its target mRNA. *FF-Luc-HLA-A2-*3′*UTR* mRNA levels were analysed by qRT–PCR. Results are relative to siControl and expressed as mean±s.d. of three independent experiments (*n*=3). **P-value <0.005 versus siCTRL+MEX-3C; NS, not significant; unpaired Student’s *t*-test (Supplementary Fig. 2c shows validation of knockdowns).

We wanted to determine which deadenylase subunit was responsible for the mRNA degradation, and therefore used siRNAs to deplete cells of deadenylase components. MEX-3C bound both the CCR4-NOT and PAN2-PAN3 deadenylation complexes in HEK293T cells. Despite effective depletion of all deadenylase components (Supplementary Fig. 2c), only the siRNA-mediated depletion of CNOT7/8(Caf1), a component of the CCR4-NOT complex, significantly rescued MEX-3C-mediated degradation of endogenous *HLA-A2* mRNA (Supplementary Fig. 2b) and of the reporter *FF-Luc-HLA-A2 3′UTR* mRNA^3^ (Fig. 2b). By analogy with RINGless MEX-3C, depletion of the CNOT7/8(Caf1) deadenylase subunit prevented mRNA degradation, but importantly did not affect MEX-3C’s ability to repress HLA-A2 translation (Fig. 2a and Supplementary Fig. 2a). The *FF-Luc-HLA-A2 3′UTR* reporter system^3^ was used in this experiment as we previously showed it reduces the bias seen with endogenous HLA-A2 at the transcriptional level^3^ and following mRNA maturation. Although depletion of CNOT7/8(Caf1), and not CNOT6/6L(CCr4), rescued MEX-3C-mediated degradation, this was not surprising as each deadenylation subunit (CNOT7/8(Caf1) or CNOT6/6L(CCr4)) regulates the expression of distinct groups of mRNAs with little overlap^10^. Previous studies have shown that only CNOT7/8(Caf1) knockdown cells showed a pronounced defect in P-body formation^9^.

### MEX-3C’s E3-ligase activity regulates deadenylation

The above results are reminiscent of the two-step miRNA-mediated repression of mRNAs by the CCR4-NOT complex, which requires an initial translational repression step, followed by the degradation of the target mRNA by deadenylation^11^. As MEX-3C interacts with Argonaute proteins^5^, which play a key role in RNA silencing, we asked whether MEX-3C’s E3-ligase activity regulates the transition between these two steps by triggering deadenylation.

**Figure 3:**
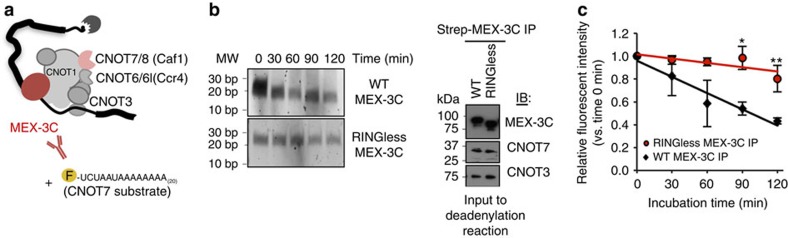
MEX-3C’s E3 ubiquitin-ligase activity regulates deadenylation. (**a**) Schematic of deadenylation assay. (**b**) Deadenylation time course. Strep-MEX-3C pull-downs were incubated with a 5′-fluorescein-labelled specific RNA substrate (Flc-5′-UCUAAAUA_20_) to assay the deadenylation activity of CNOT7 over the indicated time points. Degradation of 5′-fluorescein-labelled RNA substrate was visualized by denaturing polyacrylamide gel electrophoresis. Right panel shows loading controls for the assay. (**c**) Quantification of the substrate's fluorescence intensity remaining after deadenylation reaction. Results are expressed as mean±s.d. of three independent experiments. *P<0.05 and **P<0.01, both versus WT of the same time point; unpaired Student’s *t*-test. IB, immunoblot.

We therefore set up an *in-vitro* deadenylation assay using a specific (fluorescein labelled) RNA substrate for CNOT7 (ref. 12). Strep-MEX-3C pull-downs from wild-type (WT) and RINGless MEX-3C-expressing cells were incubated with a 5′-fluorescein labelled specific RNA substrate (Flc-5′-UCUAAAUA_20_) to assay the deadenylation activity of CNOT7 over time. Degradation of 5′-fluorescein-labelled RNA deadenylation substrate was visualized by denaturing polyacrylamide gel electrophoresis. The deadenylation substrate was readily degraded following WT MEX-3C IP, an effect not seen with RINGless MEX-3C (Fig. 3b,c). These results confirm that MEX-3C’s E3-ligase activity is required for the deadenylation of its substrate.

### Ubiquitination of CNOT7 regulates its deadenylation activity

**Figure 4:**
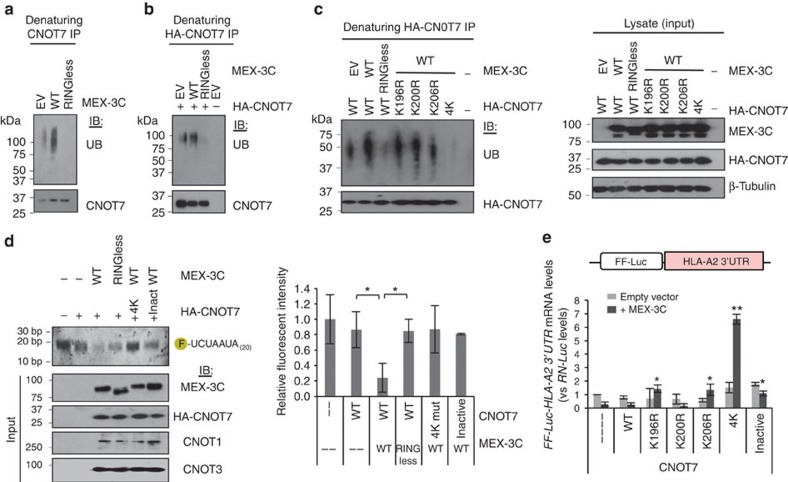
Ubiquitination of CNOT7 by MEX-3C is required for its deadenylation activity. CNOT7 is ubiquitinated *in vivo* in a MEX-3C-dependent manner. Ubiquitin blots following denaturing immunoprecipitation (IP) of endogenous (**a**) or overexpressed (**b**) CNOT7. IB, immunoblot; EV, empty vector (Supplementary Fig. 3a,b shows expression/loading controls). (**c**) Identification of the lysine residues in CNOT7 ubiquitinated by MEX-3C *in vivo*. 4K-mutant: K196R,K200R,K203R,K206R. (**d**) Ubiquitination of CNOT7 by MEX-3C is required for its deadenylation activity. Deadenylation assay was performed as described in Fig. 3. *P<0.05; unpaired Student’s *t*-test. (**e**) CNOT7 4K-mutant rescues MEX-3C-mediated degradation of *FF-Luc-HLA-A2 3′UTR* reporter mRNA *in-vivo*. *FF-Luc-HLA-A2-*3′*UTR* mRNA levels were analysed as in Fig. 2. CNOT7 catalytic inactive mutant (Inact): D40A/E42A. *P<0.05 and **P<0.005, both versus wtCNOT7+ MEX-3C treatment; unpaired Student’s *t*-test. Results are expressed as mean±s.d. of three independent experiments and relative to EV control (Supplementary Fig. 4c shows expression/loading controls for this figure).

Since Caf1 (CNOT7/8) is the major catalytic component of the CCR4-NOT deadenylation complex, and was the only deadenylation subunit whose depletion prevented MEX-3C-mediated degradation of *FF-Luc-HLA-A2 3′UTR* mRNA, we determined whether CNOT7 was a ubiquitination target of MEX-3C. Endogenous or overexpressed (HA-tagged) CNOT7 was IP under denaturing conditions to prevent interaction with other proteins. Ubiquitin immunoblot analysis showed CNOT7 ubiquitination with WT but not RINGless MEX-3C (Fig. 4a,b), confirming that CNOT7 is indeed ubiquitinated in a MEX-3C-dependent manner.

This ubiquitination did not promote CNOT7 protein degradation (Fig. 1b and Supplementary Fig. 3a,b) as CNOT7’s protein half-life was unaffected following cycloheximide treatment either in the absence (shMEX-3C) or in the presence of exogenously expressed WT or RINGless MEX-3C (Supplementary Fig. 3c).

We then wanted to determine which CNOT7 lysine residue is ubiquitinated by MEX-3C and use these findings to ask how MEX-3C-mediated ubiquitination of CNOT7 affects its deadenylation activity *in-vitro*, and the stability of the *FF-Luc-HLA-A2 3′UTR* reporter mRNA *in-vivo.* The choice of lysines to be mutated by site-directed mutagenesis was based on the structure of CNOT7 and the lysine residues (K200 and K206) reported to be ubiquitinated in mass spectrometry data sets^13^,^14^. A representation of CNOT7’s structure highlights (in blue) the exposed lysine residues mutated (Supplementary Fig. 4a). (The CNOT7 K203R variant was toxic to cells and therefore excluded from this and further experiments). In comparison to WT CNOT7, ubiquitination of the K206R mutant was mildly impaired, whereas ubiquitination was completely lost with the 4K (K196R,K200R,K203R,K206R) CNOT7 mutant (Fig. 4c).

Using the *in-vitro* CNOT7 deadenylation assay we found that the CNOT7 4K-mutant, which is no longer ubiquitinated by MEX-3C (Fig. 4c), failed to efficiently degrade CNOT7’s deadenylation substrate *in vitro* (Fig. 4d). Furthermore, overexpression of this mutant form of CNOT7 (4K-mutant) inhibited MEX-3C-mediated degradation of the *FF-Luc-HLA-A2 3′UTR* reporter mRNA *in vivo* (Fig. 4e).

To control for the structural integrity of CNOT7 4K-mutant, we performed the *in-vitro* CNOT7 deadenylation assay following CNOT7 pull-down in the absence of exogenous MEX-3C as previously described (Suzuki *et al.*)^12^. This CNOT7 4K-mutant remains functionally active in the absence of MEX-3C (Supplementary Fig. 4b) suggesting that the folding of CNOT7 4K-mutant and its ability to form a functional deadenylation complex (Fig. 4d) remained intact. Although ubiquitination is not required for CNOT7’s basal deadenylation, these results highlight that this deadenylation activity can be modulated in a novel ubiquitin-dependent manner in mammalian cells for specific mRNAs.

### A new non-proteolytic function for ubiquitin in mRNA decay

**Figure 5:**
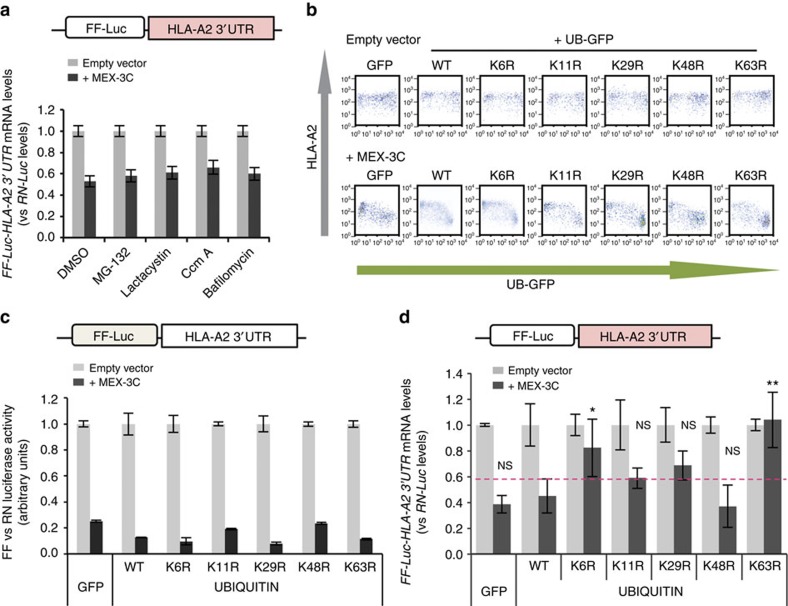
A non-proteolytic function for ubiquitin in the regulation of mRNA decay. (**a**) Neither proteasome nor lysosome inhibitors rescue MEX-3C-mediated degradation of *FF-Luc-HLA-A2-*3′*UTR* mRNA *in-vivo*. CcmA, Concanamycin A. *FF-Luc-HLA-A2-*3′*UTR* mRNA levels were analysed as in Fig. 2. Results are expressed as mean±s.d. of three independent experiments and relative to empty vector control. (**b**,**c**) Ubiquitin lysine mutants are unable to rescue MEX-3C-mediated downregulation of HLA-A2 protein levels (**b**, as determined by flow cytometry analysis) or luciferase protein levels from *FF-Luc-HLA-A2-*3′*UTR* reporter (**c**, as determined by relative luciferase activity). ‘**b**’ also serves as a control for the ubiquitin mutant (GFP) expression levels for the following experiments. (**d**) MEX-3C-mediated degradation of *FF-Luc-HLA-A2-*3′*UTR* reporter mRNA is rescued by K6R and K63R ubiquitin mutants. *P<0.05 and **P<0.005, both versus+MEX-3C+WT Ubiquitin treatment. NS, not significant; unpaired Student’s *t*-test. Results for this figure are expressed as mean±s.d. of three independent experiments and relative to empty vector control/GFP.

In addition to its role in proteolysis, ubiquitin provides a signal for a range of non-proteolytic functions^15^, by virtue of forming chains of distinct topologies depending on whether they are linked through one of its seven Lysine (K) residues or at the N-terminus^15^,^16^. Substrates modified by K48-linked polyubiquitin chains are targeted to proteasomes for degradation. In contrast, K63-linked chains provide non-proteolytic signals, as characterized in DNA damage and repair pathways, kinase signalling pathways and endocytosis^16^. Since neither proteasome nor lysosome inhibitors rescued MEX-3C-mediated degradation of the *FF-Luc-HLA-A2 3′UTR* mRNA (Fig. 5a and Supplementary Fig. 4c for expression levels), a new non-proteolytic function for ubiquitin in the regulation of mRNA decay was suggested.

To determine the ubiquitin chain linkage required for MEX-3C-mediated degradation of *HLA-A2* mRNA, we used a range of Lysine-to-Arginine Ubiquitin-Green Fluorescent Protein (UB-GFP) mutants^15^. These ubiquitin mutants are particularly useful as the co-translational cleavage of GFP from ubiquitin provides a quantitative surrogate marker for mutant ubiquitin expression^15^ (Fig. 5b). None of the ubiquitin lysine mutants rescued endogenous HLA-A2 protein levels (Fig. 5b) from MEX-3C downregulation, and this was in keeping with RINGless MEX-3C, which lacks E3-ubiquitin ligase activity^3^, and is still able to inhibit HLA-A2 translation without triggering its mRNA degradation. Similar results were obtained for firefly luciferase protein levels (measured as relative luciferase activity against renilla luciferase), when the *FF-Luc-HLA-A2-3′UTR* reporter was used^3^ (Fig. 5c).

We therefore determined the effect of the ubiquitin mutants on MEX-3C-mediated mRNA degradation. Degradation of the *FF-Luc-HLA-A2-3′UTR* reporter mRNA was rescued by ubiquitin mutants that cannot form K6- and K63-linked chains (Fig. 5d) suggesting an important role for these lysine residues in deadenylation. Furthermore, CNOT7 ubiquitination by MEX-3C was significantly reduced with the K6R and K63R ubiquitin mutants (Supplementary Fig. 4d). Conversely, neither MEX-3C-mediated ubiquitination of CNOT7 (Supplementary Fig. 4d) nor the degradation of *FF-Luc-HLA-A2-3′UTR* target mRNA was impaired in the presence of K11- or K48-linkage ubiquitin mutants, which are traditionally associated with protein degradation signals (Fig. 5d). Taken together, these results suggest a non-proteolytic function for ubiquitin in the regulation of mRNA decay by MEX-3C.

## Discussion

We have identified a new role for ubiquitin in the regulation of deadenylation, the initial and rate-limiting step in mRNA degradation. MEX-3C, a member of the recently described family of RNA-binding ubiquitin E3-ligases^4^, associates with the cytoplasmic deadenylation complexes, and ubiquitinates CNOT7, the main deadenylase subunit of the CCR4-NOT machinery. Ubiquitination of CNOT7 by MEX-3C promotes its deadenylation activity and therefore *MHC-I* mRNA degradation.

In addition to its established role in protein regulation/degradation, ubiquitination provides a critical signal for many other cellular regulatory functions^15^ and here, we have uncovered a new non-proteolytic role for ubiquitin in the regulation of mRNA decay. Ubiquitination of CNOT7 did not lead to its degradation (Fig. 1b and Supplementary Fig. 3a–c) or did proteasomal or lysosomal inhibitors rescue MEX-3C-mediated degradation of its target mRNA (Fig. 5). Degradation of the target mRNA was, however, rescued in the presence of ubiquitin mutants that cannot form K6- and K63-linked chains, presumably due to reduced ubiquitination of CNOT7 (Supplementary Fig. 4d). These results add a new signalling function to K6- and K63-linked ubiquitin chains. K6-linked chains have recently been associated with parkin’s regulation of mitochondrial quality control^17^ and to the stabilization of RING1b^18^ and BRCA1/BARD^19^, both involved in histone modification and DNA repair. K63-linked chains mediate different processes including endocytosis, assembly of DNA repair complexes and the activation of the nuclear factor-κB pathway. In fact, MEX-3C was recently reported to activate the nuclear factor-κB pathway by ubiquitinating RIG-I after viral infection in a K63-linked manner^20^.

Ubiquitination of CNOT7 was not a prerequisite for basal deadenylation activity. However, the loss of specific mRNA degradative activity with the CNOT7 4K-mutant, highlights a novel role for ubiquitin in regulating deadenylation of certain mRNAs in the mammalian system. This is important since the mechanisms that regulate deadenylation in mammalian cells are highly regulated but poorly understood. Deadenylation and RNA turnover play an important role in a broad range of cellular conditions including development, mRNA surveillance, DNA damage, cell differentiation and cancer^8^. Understanding how ubiquitin regulates mRNA abundance and protein production will provide a better mechanistic understanding of different disease states.

A number of potential mechanisms may account for MEX-3C’s ability to regulate mRNA decay through ubiquitination. Ubiquitin may induce conformational changes in CNOT7 that activate deadenylation. Alternatively, ubiquitinated CNOT7 provides a scaffold to recruit accessory proteins for activation of the degradation machinery. A similar ubiquitin-mediated regulation has been observed in the activation and disassembly of the spliceosome at distinct steps of the splicing reaction^21^.

MEX-3C is not the only ubiquitin E3-ligase to bind and regulate RNA, but belongs to a family of at least 15 RNA-binding proteins with ubiquitin ligase activity. Another prominent member of this family is CNOT4, itself a component of the CCR4-NOT deadenylation complex, and has been best studied in yeast where its orthologue (Not4) has multiple functions. These include nuclear transcriptional regulation, mRNA maturation and quality control^22^, co-translation protein quality control^23^ and proteasome assembly^24^. Not4’s contribution to mRNA deadenylation by Ccr4 (yeast homologue of CNOT6) and Caf1 (yeast homologue of CNOT7/8) is unclear. By analogy to MEX-3C, CNOT4 may also play a role in activation of the deadenylases in the CCR4-NOT complex and mRNA degradation.

Previous studies^25^ had suggested a link between ubiquitination and mRNA decay in the turnover of some AU-rich (ARE) mRNAs. Overexpression of deubiquitylating enzymes of the UBP family prolonged the half-life of specific ARE-mRNAs^25^. Furthermore proteasome inhibition prevented the rapid turnover of ARE-mRNAs, without altering the stability of non-ARE mRNAs. This latter result is in agreement with our data as *HLA-A* mRNA lacks AREs. Together these results suggest a differential role for ubiquitin in the regulation of mRNAs and highlight the diversity of this system. It will be critical to further dissect the mechanisms responsible for these different types of regulation. Further studies on MEX-3C and the ubiquitin-dependent regulation of its mRNA substrates will therefore provide an excellent platform to delineate how ubiquitin controls mRNA degradation. It is remarkable that the role of ubiquitin now extends beyond protein degradation to include the regulation and turnover of nucleic acids.

## Methods

### Cells, plasmids and transfections

HEK293T cells were grown in RPMI-1640 medium supplemented with 10% FCS. Cells were transfected using 293-Transit Reagent (Mirus Bio) and analysed by flow cytometry or immunoblotting at 48 or 72 h following transfection.

The Streptag-His-MEX-3C and myc-MEX-3C proteins and the FF-Luc-HLA-A2-3′UTR reporter are as previously described^3^. The UB-GFP mutants are as described by Boname J.M. *et al.*^15^. The pCMV5-HA-CNOT7 construct is previously described^10^. The Lysine mutant forms of CNOT7 were made by site-directed mutagenesis as described in Mittal S. *et al.*^10^.

For the luciferase quantitative PCR assays, an TK *Renilla* luciferase reporter (pRL-TK) gene was co-transfected at a 1:20 ratio to provide an internal control. All assays were performed in triplicate, with the Renilla-luciferase control used to standardize transfection efficiency. Results are relative to control levels (set as 1), and expressed as the mean±s.d. of at least three independent experiments.

siRNA-mediated depletion in HEK293T cells was performed using Oligofectamine (Invitrogen) at 75 nM final concentration and following the manufacturer’s guidelines. The siRNAs used were ON-TARGET plus pools of four from Dharmacon: MEX-3C (RKHD2; LU-006989-00-0002), CNOT7 (CAF1; LU-012897-00-00022); CNOT6L (CCR4; LU-016411-00-0002), CNOT6 (LU-019101-00-0002), CNOT8 (LU-018791-00-0002), PAN2 (LU-021192-00-0002), DCP1A (LU-021242-00-0002). MEX-3C depletion using shRNAmir against MEX‐3C (shMEX-3C) was as previously described^3^. Mock knock-downs (siCONTROL) were performed using RISC-free Universal Control (Sigma). Cells were cultured for 60 h and then assayed by FACSCalibur (BD) or quantitative reverse transcription (qRT–PCR).

### IP and immunoblotting

For IPs, cells were lysed 72 h post transfection in 1% NP-40 in Tris-Buffered Saline (TBS) with 1 μM ZnCl_2_, 0.5 mM phenylmethyl sulphonyl fluoride (PMSF), 10 mM iodoacetamide (IAA) and Roche complete protease inhibitor for 30 min on ice. Strep-His-tagged proteins were IP with Streptactin sepharose beads (IBA GmbH) for 2 h. After three washes in lysis buffer, samples were eluted in SDS sample buffer (10 min at 98 °C). (For myc-tagged MEX-3C proteins, IPs were done as previously described in Cano *et al.*^3^) IP proteins were then separated by SDS–PAGE, and transferred to polyvinylidene difluoride (Millipore) for immunoblotting. The membranes were blocked for 1 h at room temperature, and incubated with primary antibodies overnight at 4 °C in PBST containing 5% milk. Antibodies used were: rabbit polyclonal anti-RKHD2 (MEX-3C; Abcam) used at 1:5,000 dilution and rabbit anti-CNOT1 (Proteintech), rabbit anti-CNOT7 (abN1C1, GeneTex), rabbit anti-CNOT3 (abC2C3, GeneTex), Rabbit anti-PAN2 (kindly provided by Dr Jens Lykke-Andersen, University of California San Diego, USA)—all used at 1:1,000 dilution. Rabbit Anti-PCB2 was used as negative control. Membranes were developed in West Pico Extended Chemiluminescent substrate (Thermo FisherScientific). Full images of western blots and gels are shown in Supplementary Fig. 5.

For mass spectrometry analysis of RINGless MEX-3C pull-downs, HEK293T cells were transfected with pQE empty vector (EV), WT and RINGless Strep-His-MEX-3C, lysed in 1% NP-40 buffer. Lysates were incubated with 20 U ml^−1^ RNase-I for 3 min at 37 °C and IP on Streptactin beads as described above. Co-immunoprecipitated proteins were digested with trypsin using the filter-aided sample preparation protocol and analysed by LC-MSMS. Raw spectra were processed using Proteome Discoverer 1.2 and searched against a Uniprot Human database using Mascot Daemon 2.3.2. A false-discovery rate for peptides of 0.05 was applied and reported proteins required a minimum of two peptides and a score higher than 35.

For detection of ubiquitination on CNOT7, cell lysates from 5 × 10^6^ HEK293T cells at 72 h post transfection were lysed for 30 min in 1% SDS (in TBS+1 μM ZnCl_2_, 0.5 mM PMSF, 10 mM IAA, Roche complete protease inhibitor and benzonase nuclease (Sigma)) and heated for 10 min at 85 °C to remove non-covalently bound ubiquitination before IP. Samples were then diluted tenfold in 0.1% Triton X-100 buffer (in TBS+1 μM ZnCl_2_, 0.5 mM PMSF, 10 mM IAA, Roche complete protease inhibitor) and IP for 2 h using rabbit anti-CNOT7+protein-A sepharose beads or anti-HA beads (EZview Red Anti-HA Affinity Gel, Sigma). For the blotting of ubiquitinated species, polyvinylidene difluoride membranes were incubated in 0.5% glutaraldehyde before probing with VU-1 antibody (LifeSensors) following the manufacture’s guidelines.

### Deadenylation assays

The deadenylation activity assay of purified MEX-3C complexes was adapted from Suzuki *et al.*^12^. Briefly, HEK293T cells (10^6^) were transfected with pQE (empty vector, EV), WT and RINGless Strep-His-MEX-3C and HA-CNOT7 constructs. After 72 h, cells were lysed (1% NP-40 in TBS with 5% glycerol, 1 μM ZnCl_2_, 0.5 mM PMSF and protease Inhibitors) for 30 min on ice. Strep-His-MEX-3C proteins were IP for 2 h at 4 °C using Streptactin beads. After three washes with lysis buffer, IPs were washed twice in deadenylation buffer (50 mM HEPES-NaOH, pH7.4, 150 mM NaCl, 2 mM MgCl_2_, 1 μM ZnCl_2,_ 10% glycerol, 1 mM dithiothreitol). To elute bound proteins, Streptactin beads were incubated in 20 μl of deadenylation buffer containing D-desthiobiotin (LifeTechnologies) at 5 mM (2 ×) for 60 min at 37 °C with occasional mixing. Nine microlitres of eluates were incubated with 1.5 μl of 5′-fluorescein (Flc)-labelled RNA substrate (Flc-5′-UCUAAAUA_20_) at 1 μM for 60 min (or appropriate time point) at 37 °C with occasional mixing. Reactions were stopped by adding 12 μl TBE/Urea RNA sample buffer (Bio-Rad) and heated for 3 min at 85 °C. Reaction products were separated using 7 M urea/15% polyacrylamide gel (Bio-Rad) electrophoresis and stained with SYBR Green-II RNA Gel stain (Molecular Probes). The intensity of the remaining RNA down each lane was measured using ChemiDoc MP Gel System and ImageLab 4.1 software (Bio-Rad).

### Poly(A) tail-length (PAT) assay

The PCR-based poly (A) tail assay was conducted using the Poly(A) Tail-Length Assay Kit from Affymetrix and following the manufacturer’s conditions. Total RNA was isolated from FACS-sorted MEX-3C-expressing cells^3^ using RNeasy kit (Qiagen). The specific upstream primer sequence for *HLA-A2* mRNA is PAT-Fwd2: 5′-TGCATGTGTCTGTGTTCGTG-3′, and the downstream primer A2-3UTR-Rev 5′-ATCTTCTAGATTTAATAGGGAAGGAAGAAGTTACAGC-3. The universal reverse and actin 3′UTR primers were provided by the kit. PCR reactions were performed in 20 μl containing 1 × PCR buffer, 0.4 μM each primer, 0.5 U Taq DNA polymerase and 100 ng of cDNA. The amplification protocol was: 2 min at 95 °C, followed by 33 cycles of 15 s at 95 °C, 30 s at 58 °C and 30 s at 72 °C, and was completed by a final extension of 5 min at 72 °C. PCR products were electrophoresed on 2.0% agarose gel, stained with ethidium bromide and visualized by exposure to ultraviolet light.

### RNA extraction and qRT–PCR analysis

Total RNA was extracted using the RNeasy Plus kit (Qiagen). Total RNA (2 μg) was reverse transcribed into cDNA using a poly(d)T primer and Super RT reverse transcriptase (HT Biotechnology Ltd.) following the manufacturer’s instructions. Real-time qRT–PCR was performed using the ABI Prism 7700HT Sequence Detector Systems (Applied Biosystems) and SYBR Green Master mix kit (Applied Biosystems). Briefly, all reactions were performed with 120 ng of cDNA, 12.5 μl of SYBR GREEN PCR master mix and 0.2 μM forward and reverse primers in a final reaction volume of 25 μl. Cycling parameters were 95 °C for 10 min, followed by 40 cycles of 94 °C for 30 s, 58 °C for 1 min.

*Firefly* and *Renilla* luciferase primers and PCR conditions were as described in Cano *et al.*^3^. RT–PCR primer sequences are as follows: MEX-3C-Fwd: 5′-TGAACGGGGAGCAGGCG-3′, MEX-3C-Rev:5′-TGACTTGGACGGTGGTTTGA-3′; CNOT7-Fwd: 5′-AGGAACTTCAACTTGGCAGTTT-3′, CNOT7-Rev: 5′-GACAACCATTTGACCCCTTCA-3′; CNOT6-Fwd: 5′- CCTGACCCTCGGAGGATGTAT-3′, CNOT6-Rev: 5′- GCTTGGCAATGTCTGAAGGAA-3′; DCP-1A-Fwd: 5′-GAATGACTGTCACCGCATAGC-3′, DCP-1A-Rev: 5′-CTGAGTGCTTGGCTGTAACCC-3′; PAN2-Fwd: 5′-GTGGGTGTACCTGTTTCCGTC-3′ and PAN2-Rev: 5′-GCTCTGGATCTGCCGAATATCA-3′. GAPDH was used as an internal control to normalize the difference in the amount of input cDNA. GAPDH primers used were as follows: GAPDH-Fwd: 5′-ATGGGGAAGGTGAAGGTCG-3′ and GAPDH-Rev: 5′-CTCCACGACGTACTCAGCG-3′.

### Flow cytometry

Cells were stained with mAb BB7.2 (anti-HLA-A2) primary antibody in PBS+5% FCS and visualized with goat anti-mouse Cy5-conjugated secondary antibody (Jackson ImmunoResearch Laboratories). Cells were fixed in PBS with 1% paraformaldehide (PFA), read on a FACSCalibur (BD) and analysed in FlowJo.

### Proteasome and lysosome Inhibition

HEK293T cells were transfected with either pQE empty vector (EV) or WT and Strep-His-MEX-3C, together with the FF-Luc-HLA-A2 3′UTR and Renilla luciferase reporter (pRL-TK, at a 1:20 ratio). After 48 h transfection, cells were incubated for 4 h before lysis with 40 μM MG-132, 10 μM Lactacystin, 100 nM Concanamycin A or 200 nM Bafilomycin. *FF-Luc-HLA-A2-*3′*UTR reporter* mRNA levels were analysed by qRT–PCR and normalized to *Renilla-Luc*, to standardize for transfection efficiency.

## Additional information

**How to cite this article:** Cano, F. *et al.* A non-proteolytic role for ubiquitin in deadenylation of *MHC*-I mRNA by the RNA-binding E3-ligase MEX-3C. *Nat. Commun.* 6:8670 doi: 10.1038/ncomms9670 (2015).

## Supplementary Material

Supplementary Information Supplementary Figures 1-5 and Supplementary Table 1
