# Supplementary material for: A non-proteolytic role for ubiquitin in deadenylation of MHC-I mRNA by the RNA-binding E3-ligase MEX-3C
Source: Nat Commun. 2015 Oct 16;6:8670. doi: 10.1038/ncomms9670 (PMC4617606; doi:10.1038/ncomms9670)
Supplement: Supplementary Information — Supplementary Figures 1-5 and Supplementary Table 1 [file ncomms9670-s1.pdf]

Supplementary Information: Figures & Table

- **Supplementary Figures**

Supplementary Fig. 1

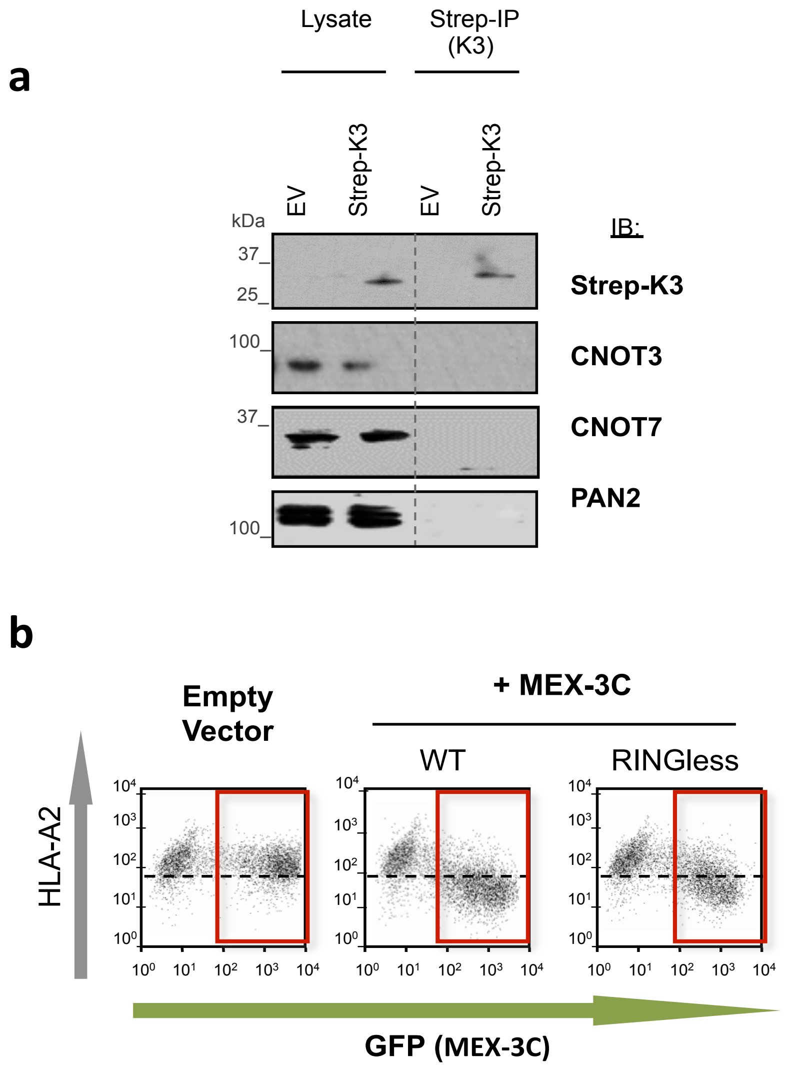

Supplementary Fig. 1:

a, Pull-down (IP) of Strep-tagged K3, an unrelated E3 ligase from KSHV, serves as a non-specific control for mass spectrometry experiments (**Figure 1a-b**). EV: Empty vector.

b, Flow cytometric analysis of HEK293T cells expressing Empty Vector (+GFP) control, WT or RINGless MEX-3C. (GFP is used as a surrogate marker for MEX-3C expression).Figure controls for MEX-3C expression and depicts FACS sorted population (red box) for **Figure 1c**.

Supplementary Fig. 2

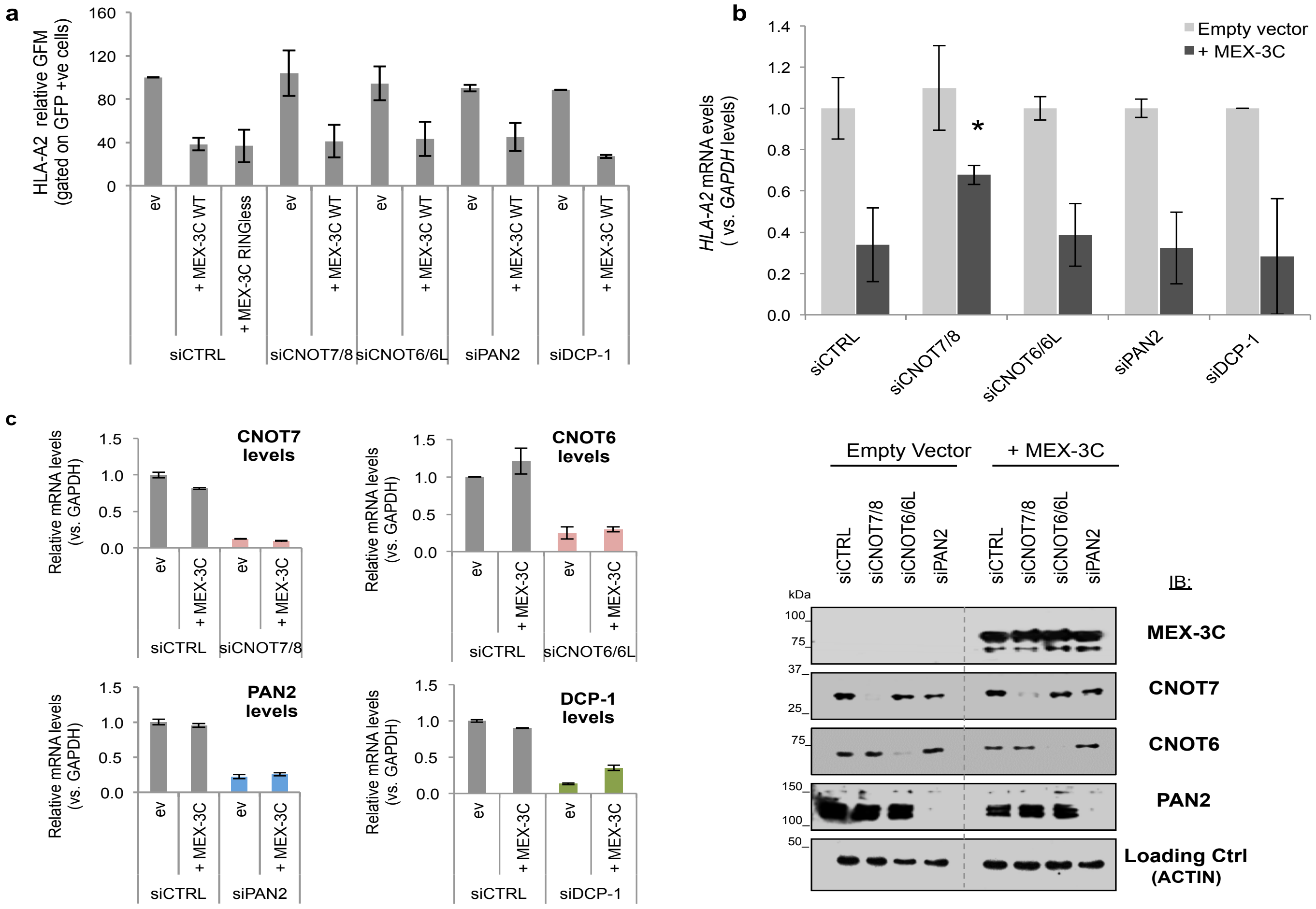

Supplementary Fig. 2:

a, Quantification of HLA-A2 geometric fluorescent means (GFM) in siRNA-treated HEK293T cells expressing wtMEX-3C (or empty vector control). GFP acts as a surrogate marker for MEX-3C expression. Figure refers to **Figure 2a**.

b, Depletion of CNOT7/8(Caf1) rescues MEX-3C-mediated degradation of endogenous *HLA-A2* mRNA levels, as analysed by qRT-PCR. Results are relative to siControl and expressed as mean ± SD of 3 independent experiments. *: $P<0.05$ vs. + MEX-3C + siCONTROL.

c, Efficacy of the siRNA knockdowns in **Figure 2**, assessed by qRT-PCR (left panel) and western blot (right panel) .

Supplementary Fig. 3

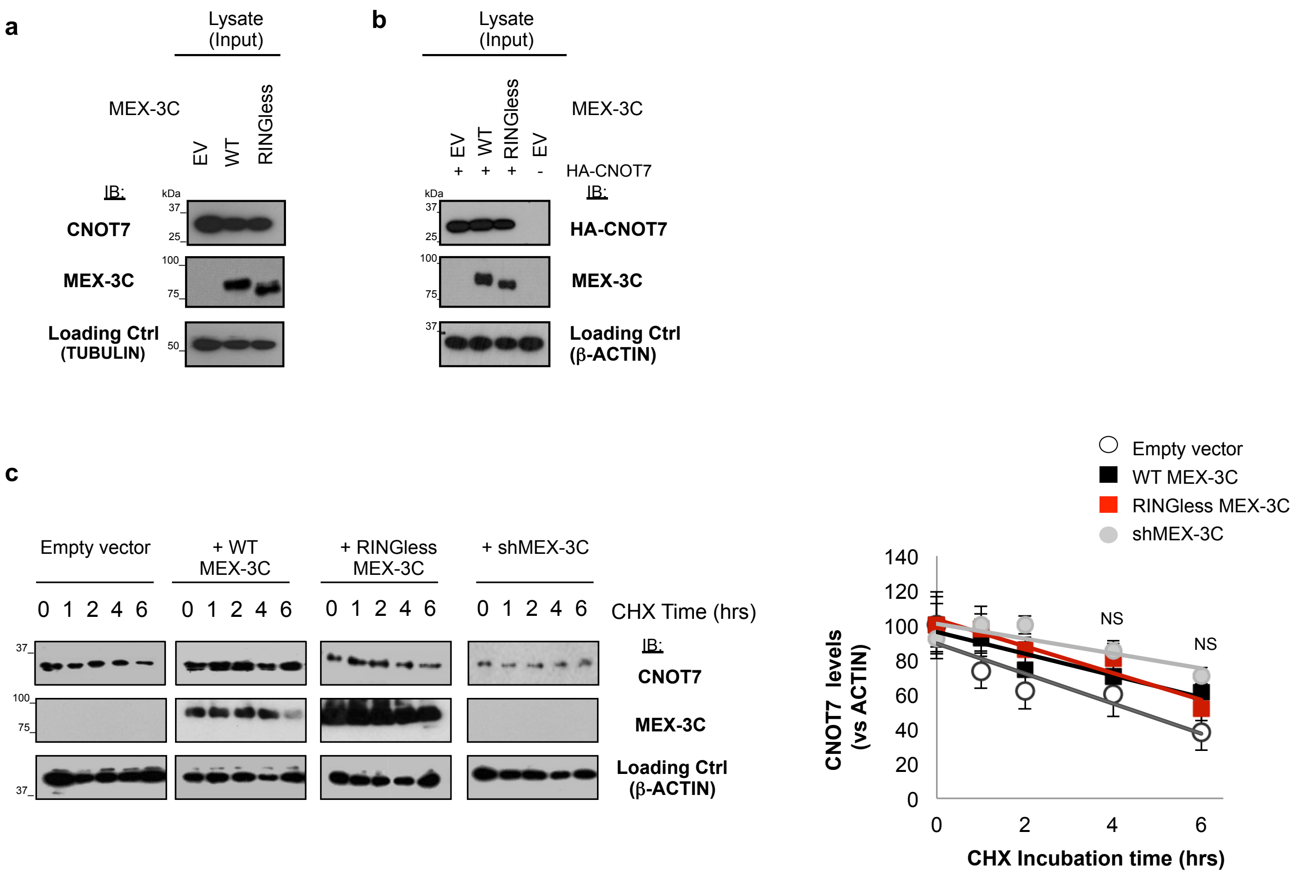

Supplementary Fig. 3:

a-b, Immunoblot analysis of MEX-3C and CNOT7(Caf1) in HEK293T cells expressing the different tagged constructs. The blots refer to input (lysate) protein levels of **Fig. 4a-b.** **c,** MEX-3C's E3 ligase activity does not affect CNOT7 protein half-life. HEK293T cells were incubated with Cycloheximide (CHX) at 100 µg/ml for 0, 1, 2, 4 and 6 hours. **Left Panel** shows immunoblot (IB) analysis of MEX-3C, CNOT7 (endogenous) and ACTIN levels after CHX treatment. Neither depletion (shRNAmir against MEX-3C, shMEX-3C) nor overexpression of WT or RINGless MEX-3C affected the half-life of CNOT7. **Right Panel:** Quantification of CNOT7's protein levels after CHX treatment. Results are expressed as % of input levels (at Time 0 hrs) and relative to loading control (ACTIN) levels. Results are the mean ± SD of three independent experiments. NS: not statistically significant.

Supplementary Fig. 4

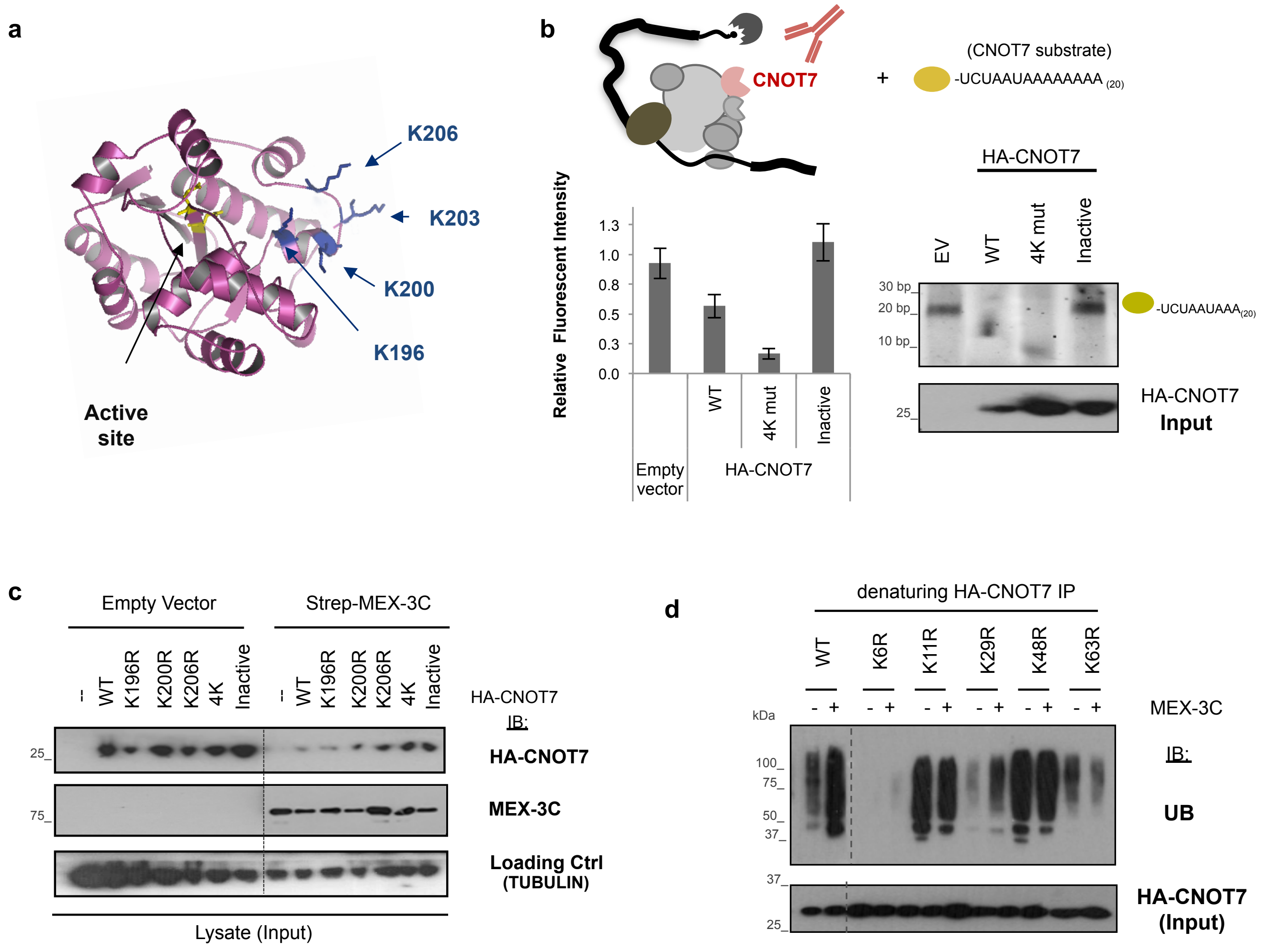

Supplementary Fig. 4:

a, Diagramatic representation of CNOT7(Caf1)'s structure[11] highlighting in blue the lysine residues mutated in **Figure 4**. Residues in active site (mutated in catalytic inactive mutant) are shown in yellow.

b, Deadenylation assays for CNOT7(Caf1) mutants, in the absence of MEX-3C. Inactive: CNOT7 catalytic inactive mutant D40A/E42A. Assays were performed as described in Suzuki et al., 2010[10]. Briefly, eluates from HA-CNOT7 pull-downs were incubated with a 5'-fluorescein labelled specific RNA substrate (Flc-5'-UCUAAAUA$_{20}$) to assay the deadenylation activity of CNOT7. Degradation of 5'-fluorescein-labelled RNA substrate was visualised by denaturing polyacrylamide gel electrophoresis (Right Panel). (Right panel also shows loading controls for the assay). Left Panel shows quantification of the substrate's fluorescence intensity remaining after deadenylation reaction. Results are expressed as mean ± SD of 3 independent experiments.

c, Immunoblot (IB) analysis of MEX-3C and CNOT7(Caf1) levels in HEK293T cells expressing the different tagged constructs. The blots refer to input (lysate) protein levels of **Fig. 4e**.

d,Ubiquitin blots following immunoprecipitation (IP) of HA-CNOT7 under denaturing conditions. Cells expressing wild-type or mutant ubiquitin-GFP constructs were lysed under denaturing conditions, immunoprecipitated for HA-CNOT7 and immunoblotted for ubiquitin. IB: Immunoblot.

Supplementary Figure 5:

Figure 1 b

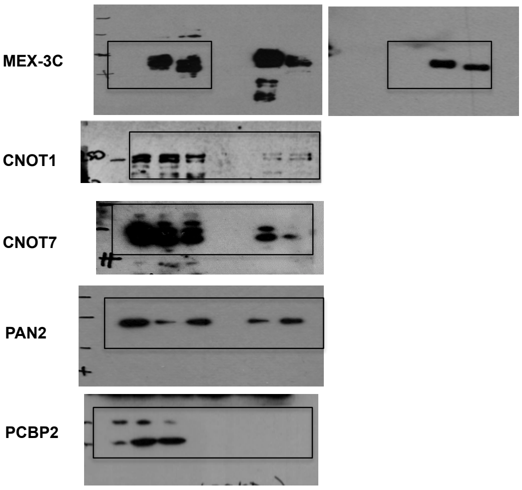

Figure 1 c

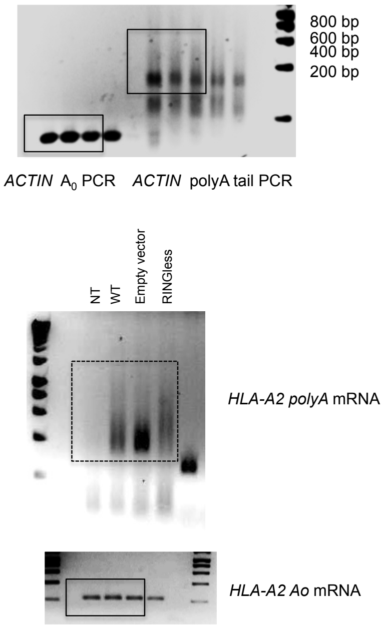

Figure 3b

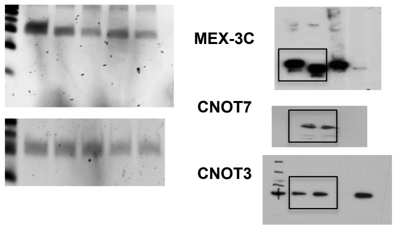

Figure 4c

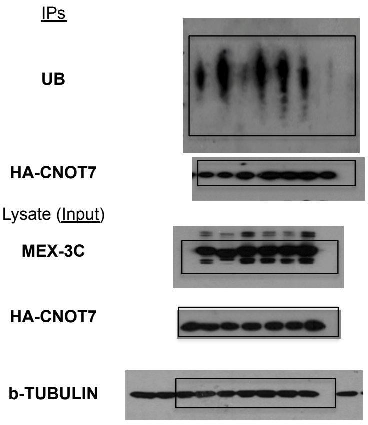

Figure 4d

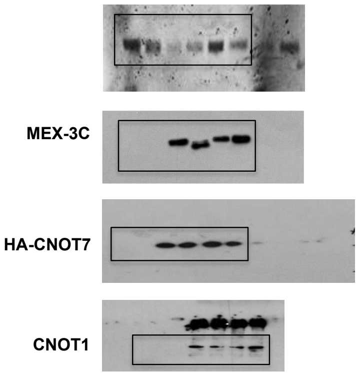

Supplementary Figure 5:

Uncropped images of Western blots and gels from Figures 1b, 1c, 3b, 4c and 4d. Antibodies used for immunoblotting are indicated next to the corresponding blot. Note: The original gels are shown. In Figure 1C, lanes 2 and 3 have been reordered in order to make it consistent with the rest of the figure.

- ## Supplementary Table 1

Mass Spectrometry analysis of Strep-RINGless MEX-3C pull-down in HEK293T cells.

Name	Description	UniProtKBAccession[a]	MW [kDa]	Score[b]
**MEX-3C**	**RNA-binding protein MEX3C**	**Q5U5Q3**	**69.3**	**7492.52**
ADAR1	Double-stranded RNA-specific adenosine deaminase	P55265	103.6	123.42
AIFM1	Apoptosis-inducing factor 1, mitochondrial	O95831	66.3	60.00
AP2A1b	AP-2 complex subunit alpha-1	O95782	105.3	146.03
AP2A2	AP-2 complex subunit alpha-2	O94973	103.9	185.35
AP2B1	AP-2 complex subunit beta	P63010	104.5	215.50
ATAD3A	ATPase family AAA domain-containing protein 3A	Q9NVI7	66.2	107.56
ATXN2L	Ataxin-2-like protein	Q8WWM7	102.8	73.04
BMS1	Ribosome biogenesis protein BMS1 homolog	Q14692	145.7	192.75
C1TC	C-1-tetrahydrofolate synthase, cytoplasmic	P11586	101.5	97.37
CAD	CAD protein	P27708	235.9	225.15
CCAR2	Protein KIAA1967; CCAR2	Q8N163	102.8	208.53
**CNOT1**	**CCR4-NOT transcription complex subunit 1**	**A5YKK6**	**266.8**	**142.47**
**CNOT3**	**CCR4-NOT transcription complex subunit 3**	**O75175**	**60.9**	**59.03**
DHX37	ATP-dependent RNA helicase DHX37	Q8IY37	129.5	79.18
DNAPK	DNA-dependent protein kinase catalytic subunit	P78527	468.8	690.12
EEF1A1	Elongation factor 1-alpha	P68104	47.8	78.29
eIF3a	Eukaryotic translation initiation factor 3 subunit A	Q14152	166.5	112.41
GIGYF2; TNRC15	GRB10 interacting GYF protein 2 isoform c	Q6Y7W6	149.4	188.92
GLE1	Nucleoporin GLE1	Q53GS7	79.8	70.67
GTPB1; GP-1	GTP-binding protein 1	O00178	72.4	93.35
HnRNPUL2	Heterogeneous nuclear ribonucleoprotein U-like protein 2	Q1KMD3	85.1	96.17
HSPA5; BiP	HSPA5 protein	P11021	72.4	515.90
ILF3; NF90; NFAR;	Interleukin enhancer-binding factor 3	Q12906	76.5	88.78
LENG8	Leukocyte receptor cluster member 8	Q96PV6	88.1	69.84
LRPPRC	Leucine-rich PPR motif-containing protein, mitochondrial	P42704	157.8	78.96
MARK3; C-TAK1	MAP/microtubule affinity-regulating kinase 2 isoform f	P27448	79.4	148.20
MRPS30	28S ribosomal protein S30, mitochondrial	Q9NP92	50.3	141.07
MYCBP2	E3 ubiquitin-protein ligase MYCBP2	O75592	509.4	396.12
MYO1C	Myosin-Ic	O00159	119.6	130.20
NOL6	Nucleolar protein 6	Q9H6R4	113.8	136.10
NUFIP2	Nuclear fragile X mental retardation-interacting protein 2	Q7Z417	76.1	117.78
NXF1	nuclear RNA export factor 1 isoform 2	Q9UBU9	40.5	97.50
POLRMT	DNA-directed RNA polymerase, mitochondrial	O00411	140.1	280.63

	precursor			
PRP6	Pre-mRNA-processing factor 6	O94906	106.9	105.25
PRPF8	Pre-mRNA-processing-splicing factor 8	Q6P2Q9	273.4	264.98
RBM19	RNA-binding protein 19	Q9Y4C8	107.3	169.91
SAFB2	Scaffold attachment factor B2	Q14151	107.4	136.44
TIF-1b; TRIM28	Transcription intermediary factor 1-beta	Q13263	88.5	158.60
WDR6	WD repeat-containing protein 6	Q9NNW5	121.6	120.92
ZAP: ZC3HAV1	Zinc finger CCCH-type antiviral protein 1	Q7Z2W4	90.2	78.88
ZNF574	Zinc finger protein 574	Q6ZN55	98.8	94.63

Supplementary Table 1:

HEK293Ts were transfected with wt or RINGless Strep-His-MEX-3C, lysed in 1% NP-40 and immunoprecipitated (IP) on Streptactin beads. Immunoprecipitations (IPs) were done in the presence of 20U/ml RNase-I. Co-IPed proteins were digested using the FASP protocol and analysed by LC-MSMS. Raw spectra were processed using Proteome Discoverer 1.2 and searched against a Uniprot Human database using Mascot Daemon 2.3.2. A peptide FDR of 0.05 was applied and reported proteins required a minimum of 2 peptides. For reported proteins the UniProtaccession[a] and Mascot score[b] are shown. Proteins with a score higher than 40 are shown.
